# Domain Segregation in Ionic Liquids Induces Long-Range Oscillatory Forces between Nanoparticles and Surfaces

**DOI:** 10.1021/acsnanoscienceau.5c00003

**Published:** 2025-05-08

**Authors:** Lívia Oliveira Xavier Silva, Kalil Bernardino

**Affiliations:** Laboratório de Química Computacional, Departamento de Química, Universidade Federal de São Carlos, Rod. Washington Luiz S/n, 13565-905 São Carlos, Brazil

**Keywords:** nanoparticle adsorption, ionic liquids, liquid crystal, oscillatory forces, molecular dynamics simulations

## Abstract

Ionic liquids have aroused great interest as solvents for the synthesis and stabilization of nanomaterials. The segregation between polar and apolar domains in ionic liquids with long alkyl groups provides kinetic stability for nanoparticle dispersions by rendering multiple free energy barriers for the aggregation. Similar effects also modulate the adsorption of nanoparticles over both liquid–vapor and liquid/solid interfaces. In this work, molecular dynamics simulations were performed to compute the potential of the mean force for the adsorption of spherical nanoparticles over solid substrates through films of imidazolium-based ionic liquids with different alkyl group lengths. While liquids with small alkyl groups produce simple profiles with barriers only close to the substrate, complex oscillatory forces arise between the nanoparticle and the substrate for ionic liquids with significant domain segregation. In addition, long-range solvent-mediated repulsive forces were also noted for liquids with an alkyl group long enough to display a smectic liquid crystal phase.

## Graphical abstract

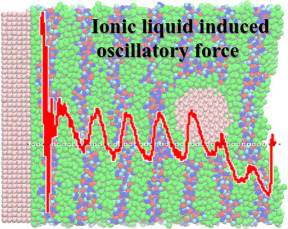

Ionic liquids (ILs) are salts with melting points below 100 °C, achieved by the presence of bulk, low-symmetry, and flexible ions.^1–3^ They are interesting as solvents for the synthesis and stabilization of nanomaterials due to their unique physical properties, including their high thermal stability, negligible vapor pressure, the ability to solvate both organic and inorganic species, high microwave absorption,^4^ and the possibility to stabilize nanoparticle (NP)^5–13^ and carbon nanotube^14,15^ dispersions without the need of additives like polymers or surfactants.

In our recent work, it was demonstrated that ILs with hydrophobic/hydrophilic domain segregation provide kinetic stability to NP dispersions although the aggregation free energy between the NPs remains favorable.^16^ The domain segregation arises in ionic liquids with large enough apolar portions (usually the cation alkyl chain) as a result of the strength of ionic interaction between the charged portions of the cation and the anion, which expels the apolar portion that interacts only by weak London forces forming a nanostructured liquid with polar and apolar regions.^17^ These domains were already observed in computer simulations^17–19^ and by light and neutron scattering experiments.^20–22^ When dispersed in ILs with domain segregation, a spherical NP induces the formation of spherical layers of IL of alternating nature depending on the chemical composition of the NP surface. A hydrophilic NP interacts better with the charged portions of the IL and induces a first layer rich in charged portions of the ionic liquid. This layer is followed by a layer of the alkyl groups, rendering an apolar region that is followed by another polar region and so on.^16^ The same trend is noticed for NPs with hydrophobic nature but with the first solvent layer being apolar. In our previous work, this was characterized for spherical NPs with 2.2 nm radius in two ILs: 1-butyl-3-methylimidazolium tetrafluoroborate (C4) and 1-octyl-3-methylimidazolium tetrafluoroborate (C8), with only the latter presenting a long enough alkyl chain to display significant domain segregation. The potential of mean force computed for the aggregation between pairs of NPs dispersed on C8 displayed a complex behavior with multiple maxima and minima that are noticeable even at distances larger than twice the NP diameter. Those oscillatory forces between NPs arise from the superposition of the solvent layers formed by each particle. The distances at which there is a superposition of solvent layers of the same nature result in a local free energy minimum. From these local minima, if either the NPs get closer or move away from each other, unfavorable superposition of solvent layers of opposite nature takes place, resulting in free energy barriers. This effect not only provides kinetic stability but also induces some long-range organization between the NPs in concentrated dispersions. Those effects are absent in the C4 liquid, which displays only a single and smaller barrier prior to NP contact.^16^

The importance of the domain segregation for the interaction between NPs dispersed in ILs raises the question of how the same effect modulates the interaction between NPs in a dispersion with solid surfaces, which is relevant to understanding how to control the deposition of NPs from an IL dispersion and also the solvent effects in top-down NP synthesis. The emergence of oscillatory forces between surfaces separated by an ionic liquid film was observed by atomic force microscopy, which demonstrated that the period of the oscillations is proportional to the length of the cation hydrophobic tail.^23,24^ The formation of alternating solvent layers was also observed in molecular dynamics simulations of thin films of ILs confined between solid surfaces.^25–29^ However, while a flat solid surface induces the formation of planar alternating layers in the IL, a small spherical NP induces the formation of concentric spherical layers. Also, the interface curvature changes the adsorption^30,31^ and, consequently, the intensity of the organization of the solvent layers.^32^ In order to explore those effects, the potential of mean force (pmf) was computed for the adsorption of a nearly spherical hydrophobic NP with a 2.2 nm radius over a flat surface of the same material across a thin film (ca. 20 nm thickness) of an IL using the umbrella sampling method.

Three ILs were selected for this study keeping the anion and the cation head fixed and changing the size of the cation tail, which modulates the occurrence and the size of the domains in the liquid: 1-butyl-3-methylimidazolium tetrafluoroborate (C4), 1-octyl-3-methylimidazolium tetrafluoroborate (C8), and 1-dodecyl-3-methylimidazolium tetrafluoroborate (C12). All the simulations were performed at 300 K using the Gromacs 2018.8 package^33,34^ with Martini 3.0 coarse grained force field^35,36^ using the same parameters and conditions as in the previous work^16^ except that no pressure coupling was used in the present work due to the presence of liquid/vapor interfaces. VMD 1.9.3 ^37^ was used to render the graphical representations. Details of the model preparation, size, interaction parameters, and potential of mean force calculation are given in the Supporting Information.

The C4 IL has a hydrophobic group that is too short and displays no significant domain segregation. Thus, the adsorption pmf in this liquid is the simplest of the studied liquids (Figure 1a). The pmf calculation started with the NP in the vapor phase just above the liquid film (*z* = 22 nm). As the NP is brought closer to the liquid surface, the attractive forces between the NP and the exposed ions lead to a large free energy decrease, indicating that the adsorption of the NP at the liquid–vapor interface is favorable for C4. The global minimum of the pmf happens when the NP center of mass is located at the liquid/vapor interface (density profile in Figure 1 b) and a repulsive force arises when bringing the NP to the interior of the liquid. The average force ⟨*F*⟩ that acts in the NP at any position *z* (bottom panel of Figure 1a) was calculated by the numerical derivative of the pmf (eq 1). The unfavorable free energy for penetration in the liquid phase is a signal of the low solubility of this hydrophobic NP in the C4 liquid, which also leads to a relatively fast aggregation between the same NPs in the bulk C4.^16^ After the NP is completely immersed in the IL (*z* = 14 nm), the pmf displays a plateau, indicating that the free energy does not change as the NP moves inside the liquid film or, equivalent, the average force acting over the NP is zero inside of the C4 film until the NP gets close to the solid (*z* < 6.5 nm). Close to the solid surface, there are two minima separated by free energy barriers. At the 5.0 nm minimum, the NP touches the solid substrate, but only with a few interaction sites. At 4.7 nm, one of the NP faces fits perfectly with the substrate crystalline structure, increasing the interaction between both and leading to a deeper minimum. Between those minima, there is a free energy barrier related to the mismatch between crystalline faces of the NP and the substrate. However, this minimum presents yet a larger free energy than the global minimum at *z* = 17.5 nm, indicating that this hydrophobic NP in C4 adsorbs stronger at the liquid/vapor than at the liquid/solid surface.

1 $$\left\langle F\right\rangle =-\frac{d(pmf\left(z)\right)}{dz}$$

**Figure 1:**
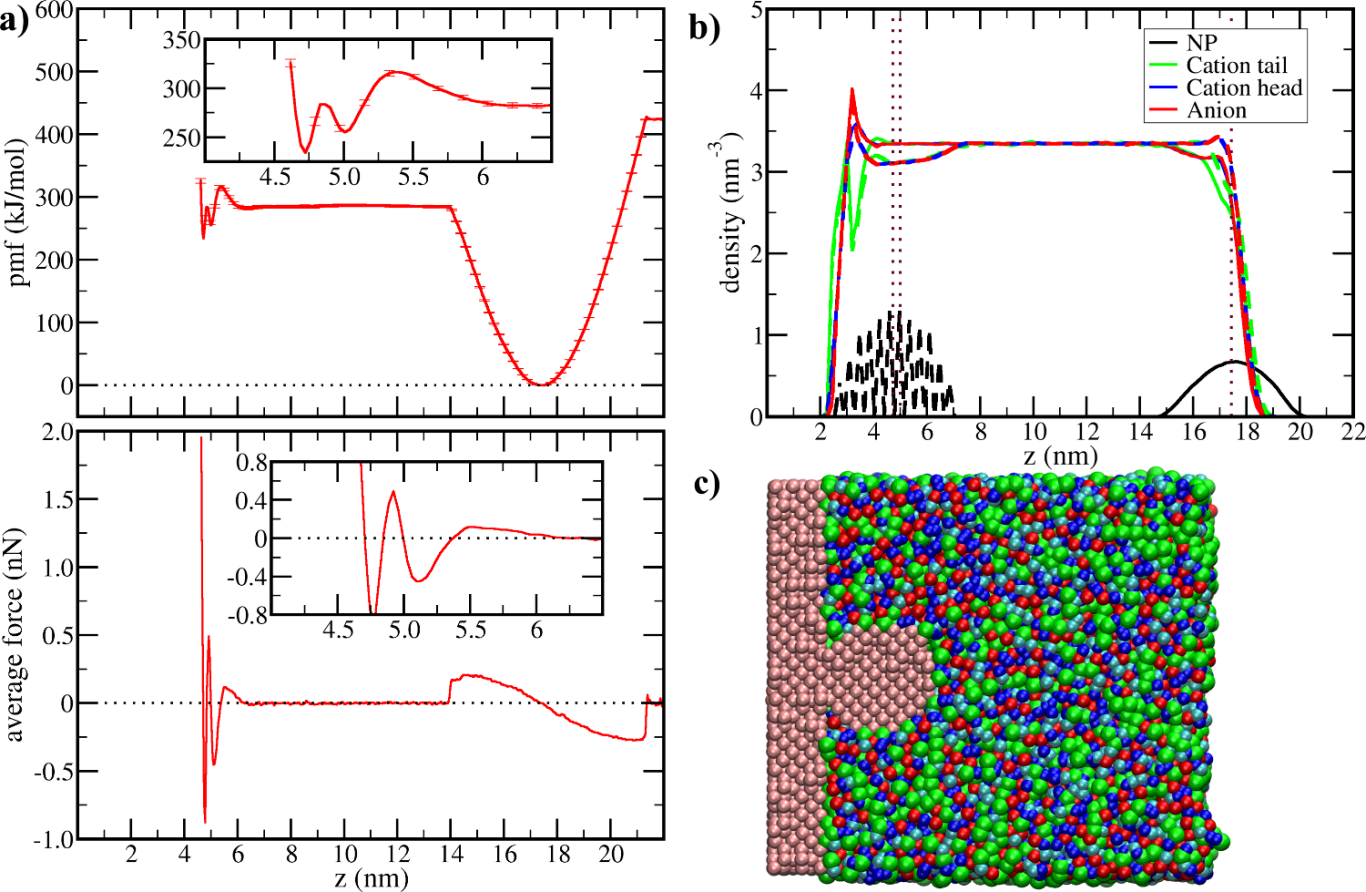
Adsorption of the NP at the solid surface from 1-butyl-3-methylimidazolium tetrafluoroborate (C4). (a) Potential of mean force (pmf) for the NP displacement in the direction perpendicular to the solid surface (top) and the corresponding average force profile (bottom). Insets zoom in on the region close to the solid surface. The solid substrate is located between *z* = 0 and *z* = 2.0 nm. (b) Number density of NP and IL sites in the direction perpendicular to the solid surface in the simulations corresponding to the pmf minimum at the liquid/vacuum interface (solid curves) and in the simulation corresponding to the minimum at the contact with the surface (dashed curves). Vertical dotted lines indicate the positions of minima in the pmf. (c) Transversal slice of the simulation box showing the NP adsorbed over the solid surface, with interaction sites of the NP and the solid shown in pink, the cation tail in green, the cation head in blue (dark blue for charged sites, light blue for uncharged), and the anions in red.

The simplicity of the pmf in C4 is related to the lack of long-range ordering of this liquid, as shown by the density profiles in Figure 1 b. The number density was computed by dividing the simulation box into slices in the direction perpendicular to the interface in two simulations for each system, with the solid curves being the results for the simulation with the NP at the liquid/vacuum interface and the dashed curves for the one with the NP at the solid/liquid interface. In both Figure 1b and in the corresponding figures for the other systems (Figures 2b and 3b), the results for the cation tail and the cation head were divided by the number of interaction sites of those species to render them in the same scale of anion density. As stated before, the structure of C4 is relatively simple, with IL species displaying a constant density except close to the interfaces with a tendency to expose the cation tails both to the vacuum and to the hydrophobic solid surface. Since C4 only shows a distinct organization at the interfaces, the pmf for the NP adsorption displays variations only at those regions. Notice also that the NP only affects the density profile of the IL by reducing them in the same slices occupied by NP interaction sites (compare solid and dashed curves), which is a trivial effect since the NP itself corresponds to an excluded volume region for the IL. Finally, the profile for the NP sites (black curves in Figure 1b) displays a continuous Gaussian shape at the liquid/vapor interface (and also at any distance before the contact with the solid) but a spiked one at the solid/liquid interface because in the former the NP can rotate and all orientations of the crystalline planes are sampled. However, once adsorbed on the solid substrate, the crystalline structure of the NP aligns with the solid substrate and can no longer rotate. This feature is present in all of the systems studied.

**Figure 2:**
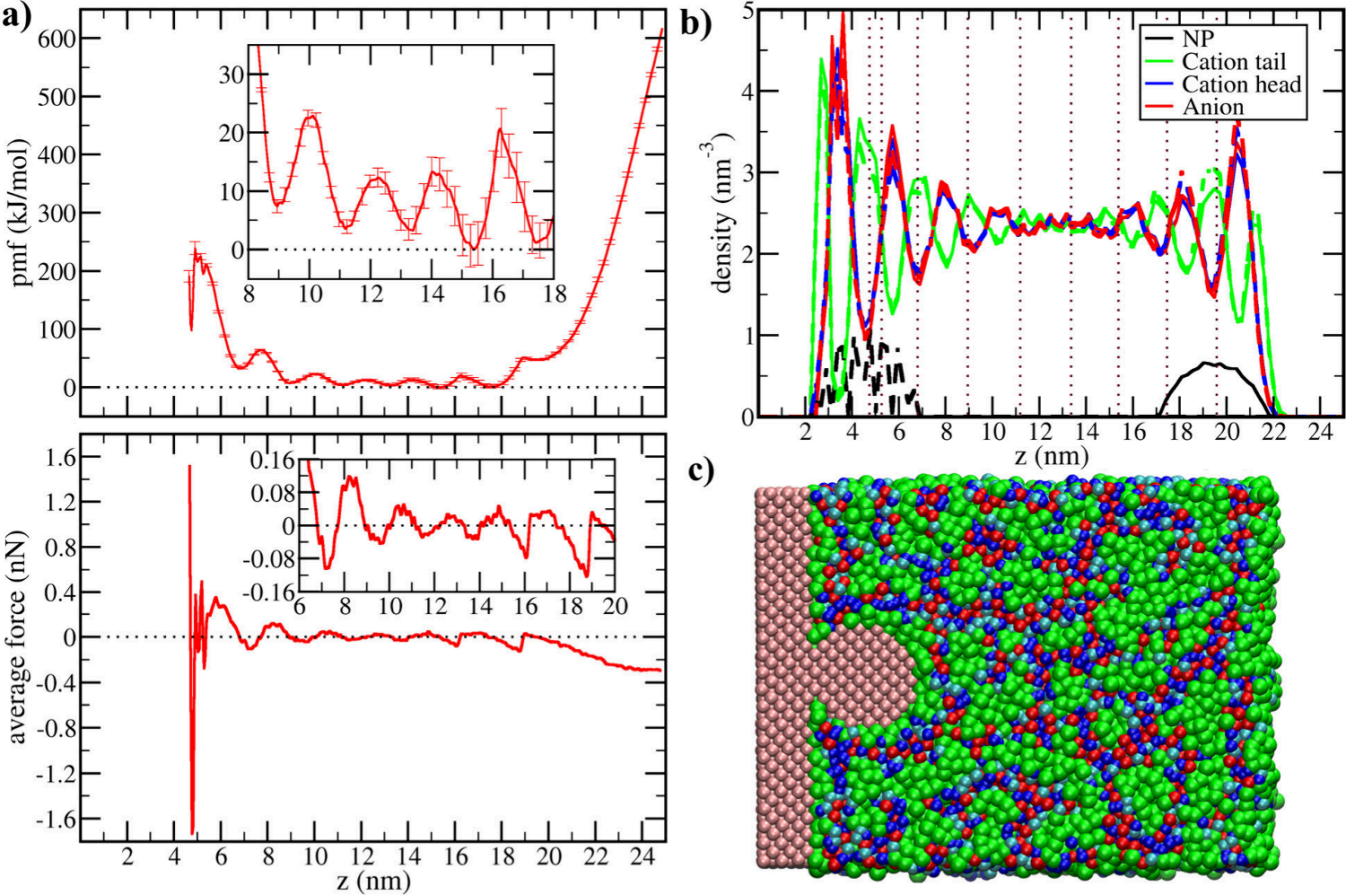
Adsorption of the NP at the solid surface from the 1-octyl-3-methylimidazolium tetrafluoroborate (C8) liquid film. (a) Potential of mean force (pmf) for the NP displacement in the direction perpendicular to the solid surface and the corresponding average force profile. Insets zoom over the middle of the liquid slab. (b) Number density of NP and IL sites in the direction perpendicular to the solid surface in the simulations corresponding to the pmf minimum at liquid/vacuum interface (solid curves) and in the simulation corresponding to the minimum at the contact with the surface (dashed curves). Vertical dotted lines indicate the positions of minima in the pmf. (c) Transversal slice of the simulation box showing the NP adsorbed over the solid surface, with interaction sites of the NP and the solid shown in pink, the cation tail in green, the cation head in blue (dark blue for charged sites, light blue for uncharged), and the anions in red.

**Figure 3:**
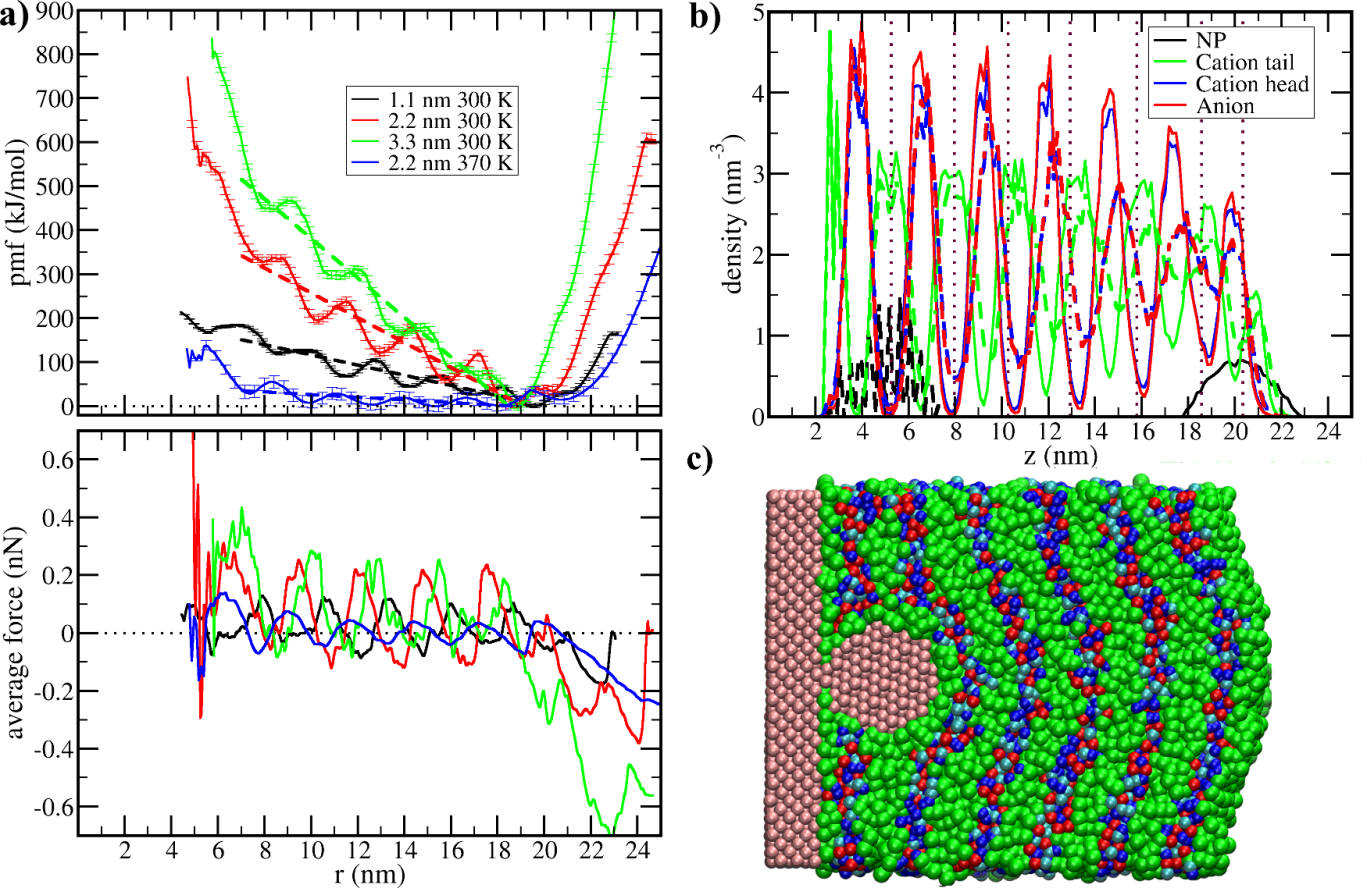
Adsorption of the NP at the solid surface from the 1-dodecyl-3-methylimidazolium tetrafluoroborate (C12) liquid crystal. (a) Potential of mean force (pmf) for the NP displacement in the direction perpendicular to the solid surface and the corresponding average force profiles, with different colors corresponding to different NP radius (1.1, 2.2, and 3.3 nm) or to different temperature (blue curve only, which corresponds to 370 K). Dashed curves correspond to linear regression between 7 and 18 nm of the corresponding pmfs. (b) Number density of NP and IL sites in the direction perpendicular to the solid surface in the simulations corresponding to the pmf minimum at liquid/vacuum interface (solid curves) and in the simulation corresponding to the minimum at the contact with the surface, both for the NP with radius 2.2 nm at 300 K. The density profiles for *T* = 370 K are given in the Supporting Information. Vertical dotted lines indicate the positions of minima in the pmf. (c) Transversal slice of the simulation box showing the NP with radius 2.2 nm adsorbed over the solid surface, with interaction sites of the NP and the solid shown in pink, the cation tail in green, the cation head in blue (dark blue for charged sites, light blue for uncharged), and the anions in red.

Increasing the length of the alkyl group in the C8 liquid leads to significant domain segregation, which results in remarkable differences in the liquid structure and adsorption pmf (Figure 2). As in the case of C4, when the hydrophobic particle is moved from the vapor phase to the liquid surface (*z* = 21 nm), a large drop is noticeable in the free energy (Figure 2a). The ability of C8 to solvate the NP better than C4 results in a further drop in the free energy as the NP is completely immersed in the liquid (*z* = 18 nm) instead of an increase.

Besides being a better solvent to disperse the hydrophobic NPs, another feature arises from the larger alkyl group in the IL: the segregation between hydrophobic and hydrophilic domains leads to liquid layers of different natures moving farther from the solid substrate. The first layer is of the same nature as the solid, in this case, hydrophobic, and is followed by a hydrophilic layer rich in cation head groups and anions, followed by a second layer rich in hydrophobic groups, and so on (Figure 2b,c). A similar organization is also noticed at the liquid/vapor interface due to the tendency of the IL to expose the weakly interacting alkyl groups to the vapor instead of the ionic portions. As a result, both interfaces generate layered structures that propagate a few nanometers inside the liquid but lose intensity as they move toward the middle of the liquid film. This structure is partially disrupted by the presence of the NP, which also tends to organize layers of the IL around itself. The interaction between the NP and the liquid layers structured by both interfaces leads to oscillations in both the pmf and the average force profiles (Figure 2a), with the local minima of the pmf corresponding to the liquid layers rich in the cation tails (Figure 2b, where vertical dotted lines correspond to the positions of the pmf minima). The liquid layers rich in the polar portions of the liquid correspond to free energy barriers larger than the thermal energy for the NP to move either toward or away from the solid surface. Similarly to the aggregation between NPs, the domain segregation in C8 leads to barriers for NP adsorption; however, while the aggregation between 2 of those NPs is thermodynamically favorable in C8, the adsorption over a solid substrate of the same material is unfavorable. Besides presenting a local minimum when in contact with the solid surface, the free energy of the adsorbed NP is higher than the free energy in the middle of the liquid film, which may be a result of a mismatch between the symmetries of the solvent layers organized by the NP and by the solid substrate. Other factors, such as the loss of both translational and rotational entropies of the NP upon adsorption, may also contribute to this difference.

C12 presents an even longer alkyl tail and, consequently, one would expect a larger free energy drop when moving from the vapor to the liquid surface as well as a larger drop when penetrating the liquid film. However, the drop when moving toward the liquid/vapor interface is nearly the same as noticed for C8 and there is a strong repulsion against the NP penetration deeper in the liquid film (Figure 3a, red curves). This happens due to a distinct structure of the C12 IL, which displays a liquid crystal behavior with a smectic phase,^38^ with a long-range structure of alternating hydrophobic and hydrophilic layers (Figure 3b,c). While C8 displays domain segregation, it is still an isotropic liquid with no long-range organization, similar to the micelles in the isotropic phase of surfactant solutions. C12, on the other hand, in temperatures below the transition to the isotropic phase, is similar to the lamellar phases observed in concentrated surfactant solutions.^39^ This leads to an even stronger organization of the liquid layers in response to the solid surface, as noted by the density profiles. As observed in C8, the NP also tends to organize the liquid in a spherical symmetry around itself and presents local minima in the pmf at the distances corresponding to the hydrophobic layers of the IL (vertical dotted lines in Figure 3b). However, the barriers between consecutive minima are higher in C12, and most notably, there is a steady increase in the free energy as the momentum moves toward the solid surface superimposed with those oscillations. A linear regression of the pmf in the region after the second polar layer and before the liquid/vapor interface (between *z* = 7 and *z* = 18 nm) was computed and showed an increase of 28 kJ/mol in the free energy for each nm the NP moves toward the solid substrate ignoring the local oscillations or a corresponding repulsive force of 0.05 nN.

The long-range repulsive force superimposed with the oscillations in the pmf expected from the layers of different natures is due to the perturbation that the NP itself introduces on the organized liquid layers of the smectic liquid crystal phase. While in the isotropic liquids (C4 and C8) the NP penetration only induces small and local perturbations in the liquid density profiles (Figures 1b and 2b), in C12 the NP disturbs every liquid layer between its position and the liquid/vapor interface, with the amplitudes of the density profile decreasing as the NP gets closer to the solid surface (compare solid and dashed line curves in Figure 3b). This effect can be better understood by looking at the average liquid structure (generated by the superposition of several simulation frames) when the NP is at several positions along the pmf (Figure 4, top). At the liquid–vapor interface, the perturbation induced in the IL lamellae is small. As the NP moves closer to the solid substrate, the layers between the NP and the liquid/vapor interface are perturbed. This results in an increase in the surface energy that gets larger as more layers of liquid are perturbed as the NP gets closer to the solid, explaining the growth of the free energy as the NP moves toward the substrate. This effect is absent in isotropic ILs even with domain segregation. Figure 4 also displays the disruption of more lamellae by the mismatch with the NP solvation layers at the distances corresponding to local maxima in the pmf than in the regions corresponding to local minima.

**Figure 4:**
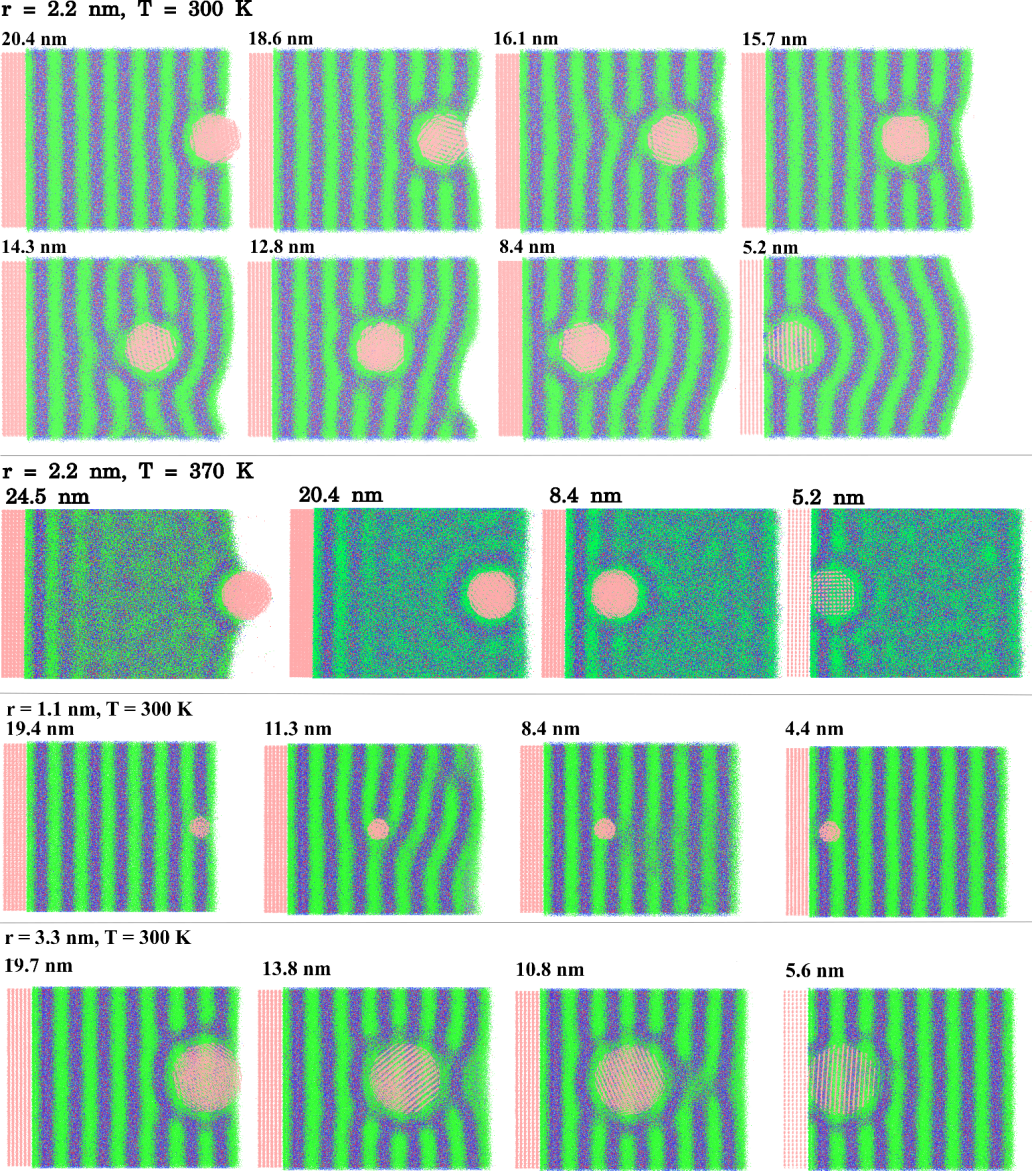
Top: transversal slice of the simulation box showing the structure of the liquid 1-dodecyl-3-methylimidazolium tetrafluoroborate (C12) at selected minima (20.4, 18.6, 15.7, 12.8, and 5.2 nm) and maxima (16.1, 14.3, and 8.4 nm) of the potential of mean force. Those representations were produced by the superposition of 50 different structures from the second half of the umbrella sampling simulations at the corresponding distances after the NP was centralized in the simulation box. NP and solid surface sites are displayed in pink, cation tail in green, cation head in blue (dark blue for charged and light blue for uncharged sites), and anions in red. Middle and Bottom: similar representations for selected distances between the NP center and the solid surface for the NP of radius 2.2 nm at C12 at a higher temperature at which a isotropic phase is observed and for the NPs of radii 1.1 and 3.3 nm at 300 K. The increase of the liquid volume in the structures displayed for 370 K is due to the thermal expansion of the IL, and the number of ions was held constant.

To better evaluate the effect of the long-range ordering of the smectic phase, additional pmf values were computed in C12. To evaluate the effect of the size, the pmf was computed for NPs with radii 1.1 and 3.3 nm (black and green curves in Figure 3a) and, to study a temperature at which the liquid became isotropic, the pmf of the reference NP with 2.2 nm radius was computed at 370 K (blue curves in Figure 3a). At 370 K, after the transition to the isotropic phase, the pmf observed for C12 becomes similar to the one for C8, without a significant long-range repulsion but with the oscillatory behavior, as there are no more ordered layers far from the solid/liquid interface to be disturbed (structures in Figure 4 and density profiles in Figure S4). The size of the particle affects the long-range repulsion, with the same increasing as the NP increases size at least in the range accessible in our calculation, with the linear regression of the pmfs between *z* = 7 and *z* = 18 nm resulting in average free energy increases of 12, 28, and 45 kJ/mol per nm as the NP moves closer to the surface for the ones with radii 1.1, 2.2, and 3.3 nm, respectively. The long-range repulsion is still significant even for the smallest NP considered besides the structural perturbation induced in the solvent being smaller and harder to notice in visual inspection (Figure 4).

The stronger perturbation induced by the NP in C12 also justifies the presence of only a shallow local minimum when the NP gets in contact with the substrate, with again the adsorption being thermodynamically unfavorable. However, a word of caution is needed: the strength of the interaction of the NP and solid surface sites as well as specific details of the surfaces, such as their roughness, will greatly affect the depth of this minimum, and depending on those factors, the adsorption may be thermodynamically favorable. Those factors must be taken into account when comparing those models with different models or experimental data.

## Summary

The interaction between nanoparticles and solid substrates through ionic liquid films strongly depends on the molecular structure of the liquid. Ionic liquids with no significant domain segregation display free energy barriers only very close to contact with the surface. The increase in the amphiphilic character achieved by longer alkyl groups in the cation leads to the formation of nanometric hydrophobic and hydrophilic domains inside the liquid, which will be organized around the nanoparticle and solid substrate. When the nanoparticle approaches the solid, the superposition between solvent layers of opposing nature established around the particle and the surface results in multiple free energy barriers and oscillatory forces that propagate for several nanometers. If the alkyl group gets even longer, the liquid becomes anisotropic, displaying many more organized polar and apolar alternating layers that propagate across the whole film, characterizing a smectic liquid crystal phase. In this phase, not only do the oscillatory forces due to the superposition of solvent layers become stronger but also an overall repulsive solvent-mediated force arises as a consequence of the long-range structure of the liquid crystal, which is perturbed in every liquid layer between the nanoparticle and the liquid/vapor interface as the particle moves closer to the solid substrate. As those liquid crystals are thermotropic, the interaction between the NP and the substrate can be controlled by the temperature. After the liquid changes from smectic to isotropic phases at higher temperatures, the long-range repulsion is eliminated, leaving only the oscillatory forces.

## Supplementary Material



## Notes

Detailed description of model system preparation, simulation conditions, and interaction parameters, histograms displaying the sampling across the reaction coordinate used for the potential of mean force calculations, and additional density profiles for the C12 liquid (PDF)
