# Supplementary material for: Domain Segregation in Ionic Liquids Induces Long-Range Oscillatory Forces between Nanoparticles and Surfaces
Source: ACS Nanosci Au. 2025 May 8;5(4):262–8. doi: 10.1021/acsnanoscienceau.5c00003 (PMC12371583; doi:10.1021/acsnanoscienceau.5c00003)
Supplement: Supplementary file 1 [file ng5c00003_si_001.pdf]

Supporting Information for

Domain segregation in ionic liquids induces long-range oscillatory forces between nanoparticles and surfaces

*Lívia Oliveira Xavier Silva, Kalil Bernardino**

* kalilb@ufscar.br

Laboratório de Química Teórica, Departamento de Química, Universidade Federal de São Carlos, Rod. Washington Luiz S/n, 13565-905 São Carlos, Brazil

Contents:

1. Model system preparation and interaction parameters 1
2. Simulation conditions 4
3. Sampling histograms of pmf calculations 7
4. Zoom over the pmfs for the C12 liquid 9
5. Additional density profiles for C12 liquid 10

1. Model system preparation and interaction parameters

Both the solid substrate and the NP were designed based on the same face-centered cubic (FCC) crystalline structure. A unit cell was draw with 4 interaction sites distant by 0.528 nm from each other (Figure S1 a). This distance corresponds to the minimum of the Lennard-Jones potential (Equation S1 and Table S1) between the interaction sites. The solid substrate was produced by replicating this unit cell 22 times in both x and y direction and 3 times in the z direction, resulting in 5808 interaction sites slab (Figure S1 b). The NP was generated by creating a large crystal based on the same unit cell and using the VMD software to extract the sites within a sphere of 2.2 nm radius, resulting in nearly spherical particles with 580 interaction sites (Figure S1 c). Only for the C12 ionic liquid, two other NP sizes were studied and those were produced similarly but extracting the sites within spheres of radius 1.1 and 3.3 nm, resulting in particles with 55 and 1481 sites, respectively.

Films of each ionic liquid were placed over the solid substrates in the z direction, being produced films with 13549 ion pairs for C4, 12050 for C8, and 9400 ion pairs for C12. Those amounts resulted, after equilibration, in liquid films of *ca.* 16 nm thickness for C4 and *ca.* 20 nm for both C8 and C12. The C4 film was made arbitrarily thinner than the others since no long-range ordering was expected for this liquid, as was observed (Figure 1 of the manuscript). The simulation boxes were elongated in the z direction to 75 nm in order to create a liquid-vacuum interface in addition to the solid/liquid interface.

After the equilibration of the liquid film in contact with the solid substrate, the nanoparticle was inserted slightly above the liquid/vacuum interface and the potential of mean force was computed to bring the nanoparticle to the solid surface though the ionic liquid film.

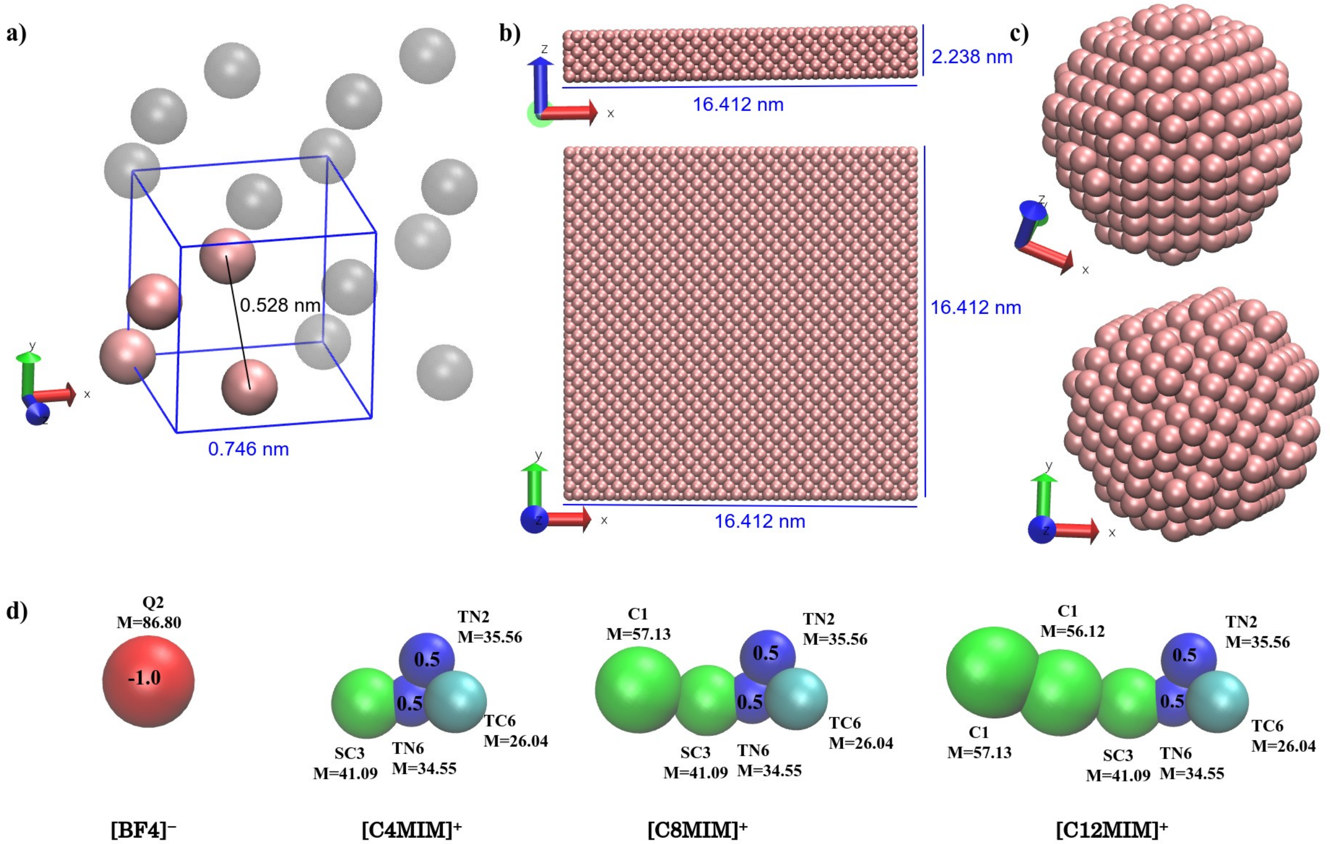

Figure S1 – Model system preparation. **a)** Unit cell used to produce the face-centered cubic (FCC) crystalline structure of both the solid substrate and the 2.2 nm radius NP, with the 4 interaction sites inside the box represented as solid pink spheres and replicas in x and y directions showed as transparent gray spheres. **b)** Structure of the solid substrate. **c)** structure of the nanoparticle. **d)** Representations of the studied ions in the coarse grained force field with the respective types and masses (in g/mol).

The models used to describe the ions in the Martini 3.0 coarse-grained force field with the respective masses, charges and types are displayed in Figure S1 d using the same colors employed in the graphical representations of the manuscript. Detailed explanation of the types used in Martini 3.0 and reported in Figure S1 can be found in the force field papers (references 35 and 36 of the

manuscript). The BF_4^- anion is described as a single negative interaction site. The imidazolium ring is described by 3 interaction sites, with the positive charge distributed between two sites (shown in blue) while the third one (in cyan) is uncharged., The cation alkyl groups are described by hydrophobic and uncharged interaction sites (green), with the difference between the 3 cations studied here being essentially the number of hydrophobic sites, with an extra site included for each 4 carbon atoms in the alkyl group. Harmonic potentials with k = 4500 kJ mol^{-1} nm^{-2} were used to describe the bonds between imidazolium ring sites while harmonic potentials with k = 2500 kJ mol^{-1} nm^{-2} were employed for bonds between alkyl group sites. Angular deformations between cation sites were also described by harmonic potentials with k = 50 kJ mol^{-1} rad^{-2} for the angles inside imidazolium ring and 30 kJ mol^{-1} rad^{-2} for the others.

Harmonic potentials with k = 2500 kJ mol^{-1} nm^{-2} were also used to describe bonds between neighbor interaction sites of the NP and with k = 50 kJ mol^{-1} rad^{-2} to describe angular deformations inside the NP. Harmonic potentials with k = 4000 kJ mol^{-1} nm^{-2} were applied over the absolute position of each site of the solid substrate to hold the crystalline structure. As the hydrophobic NP displayed the strongest effects regarding the domain segregation in the IL dispersions studied in our previous work (reference 16 of the main text), the same interaction parameters were employed here, with both NP and solid substrate sites being described by the hydrophobic type C1 of the Martini 3.0 force field. This type reproduces the interactions expected for surfaces covered with alkyl groups. The parameters for the Lennard-Jones (Equation S1) potential between the NP and the solid substrate sites with IL sites are given in Table S1.

$$U_{LJ} = 4\varepsilon\left[\left(\frac{\sigma}{r}\right)^{12} - \left(\frac{\sigma}{r}\right)^{6}\right] \quad \text{(Equation S1)}$$

Table S1 – Interaction parameters for the Lennard-Jones potential (Equation S1) between NPs interaction sites with sites from solid substrate, IL cation and IL anion (see Figure S1 for details on type attribution).

Species	Type in Martini 3.0 notation	Lennard-Jones parameters	
		ε (kJ/mol)	σ (nm)
NP or substrate sites	C1	3.390	0.470
Cation head	TC6	1.670	0.395
	TN2	1.670	0.395
Cation tail	SC3	2.920	0.430
	C1	3.390	0.470
Anion	Q2	2.370	0.520

2. Simulation conditions

All the simulations were performed at T = 300 K and τT = 1 ps using the V-rescale thermostat except one pmf performed for C12 liquid at which T = 370 K was used in order to compare the results of the smectic phase at 300 K with the profile for the isotropic phase of the same liquid, which happens only at high temperature. No pressure coupling was used due to the liquid – vacuum interfaces. The integration timestep was dt = 0.02 ps, being the use of a larger dt than the usual value of 1 fs in atomistic simulations enabled by the higher mass of coarse-grained interaction sites and smaller force constants, which results in smaller vibration frequencies than in typical atomistic simulations.

A cut-off radius of 1.1 nm was employed for non-bonded interactions with Particle-mesh Ewald (PME) correction for long-range coulomb interactions while a shift function was employed between 0.9 and 1.1 nm to make Lennard-Jones potential converges smoothly to zero at the cut-off. A relative

dielectric constant ε_r = 15.0 was employed to attenuate electrostatic interactions between charged species, in order to compensate for the lack of molecular dipoles in the coarse-grained force field.

The umbrella-sampling method was used to compute the potential of mean force (pmf). After placing the NP above the liquid film, harmonic potentials were applied between the NP and the substrate only in z direction to force the particle to sample the whole reaction coordinate until the adsorption over the substrate. The final structure of each simulation along the pmf calculation was used as the starting point for the next one, with the minimum of the external harmonic potential slightly displaced to small z values, leading to the approximation between the NP and the solid. While the NP is far from the substrate, a force constant k = 600 kJ mol^{-1} nm^{-2} was used for the bias potential and the minimum was displaced by 0.05 nm between consecutive windows. Closer to the surface (z < 7.5 nm), a larger force constant k = 5000 kJ mol^{-1} nm^{-2} was used instead due to the stronger forces acting over the NP, and the minimum was displaced by 0.02 nm instead. Even closer to the surfaces (z < 5.0 nm), k = 25000 kJ mol^{-1} nm^{-2} force constant with a window spacing of 0.01 nm was needed to properly sample local minima and maxima of the pmf which differ by the packing between crystalline structures of the NP and the solid substrate.

For the pmfs of 2.2 nm radius NP at 300 K, each simulation reached a total integration time of 20 ns, being the first 5 ns of each one discarded to guarantee the relaxation after shifting the position of the bias potential. In total, 540 sampling windows were performed for both C8 and C12 liquids and 456 for C4, resulting in total simulation times of 10800 ns for the pmf calculation of C8 and C12 and of 9120 ns for C4. The histograms showing the sampling across the reaction coordinate for the pmf calculations are given in Figure S2.

For the additional NP sizes and for the temperature of 370 K (calculations performed for C12 liquid only), the same protocol was employed, but performing only 10 ns per sampling window and discarding the first 2 ns. This reduction was tested by recomputing the previous pmfs using only the

frames between 2 and 10 ns of each sampling window and doesn't result in significant changes besides increasing the estimated error bars. The histograms for those additional pmfs are given in Figure S3.

3. Sampling histograms of pmf calculations

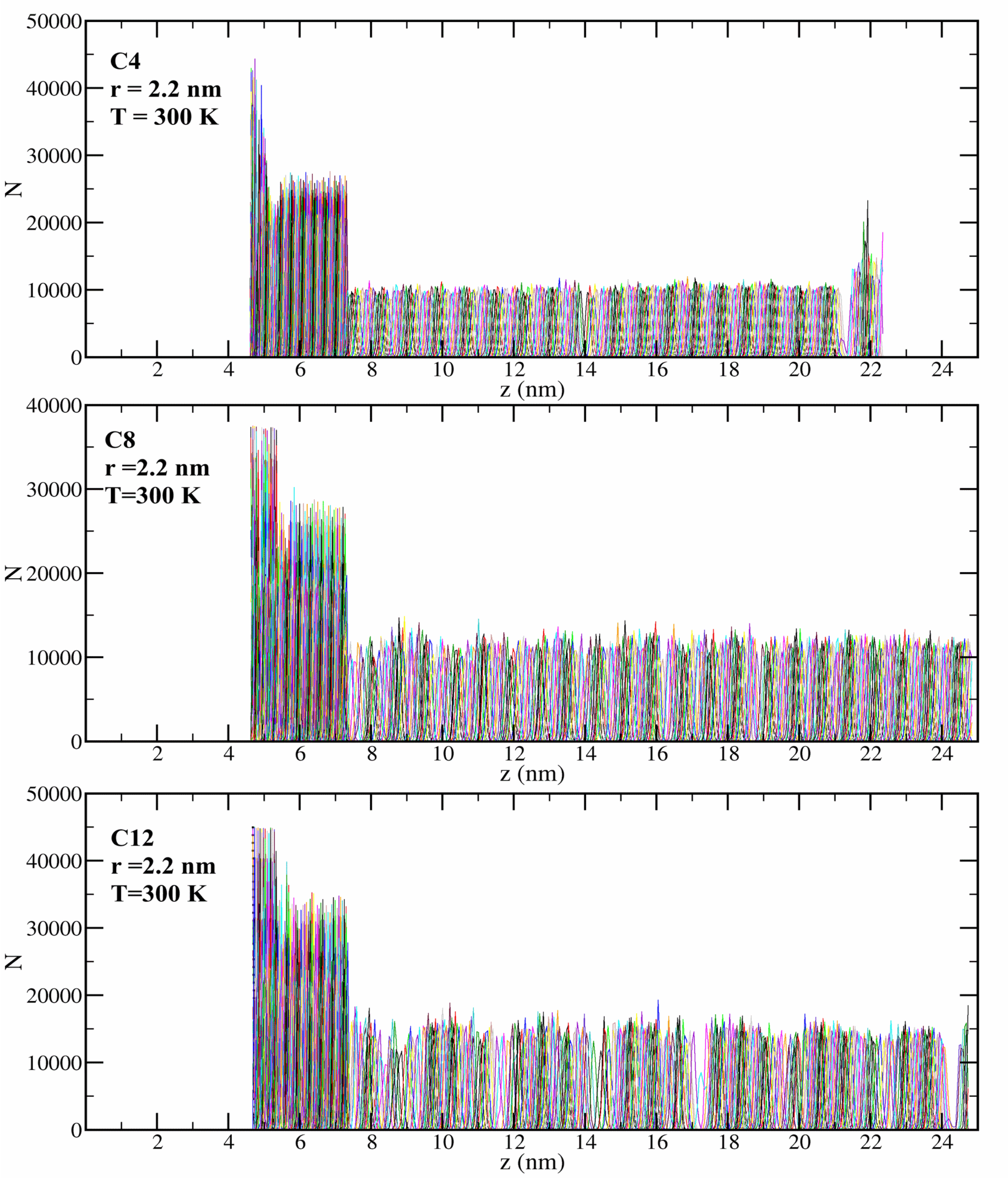

Figure S2 – Histograms showing the sampled values of the position z of the NP center of mass at each sampling window of the potential of mean force calculations.

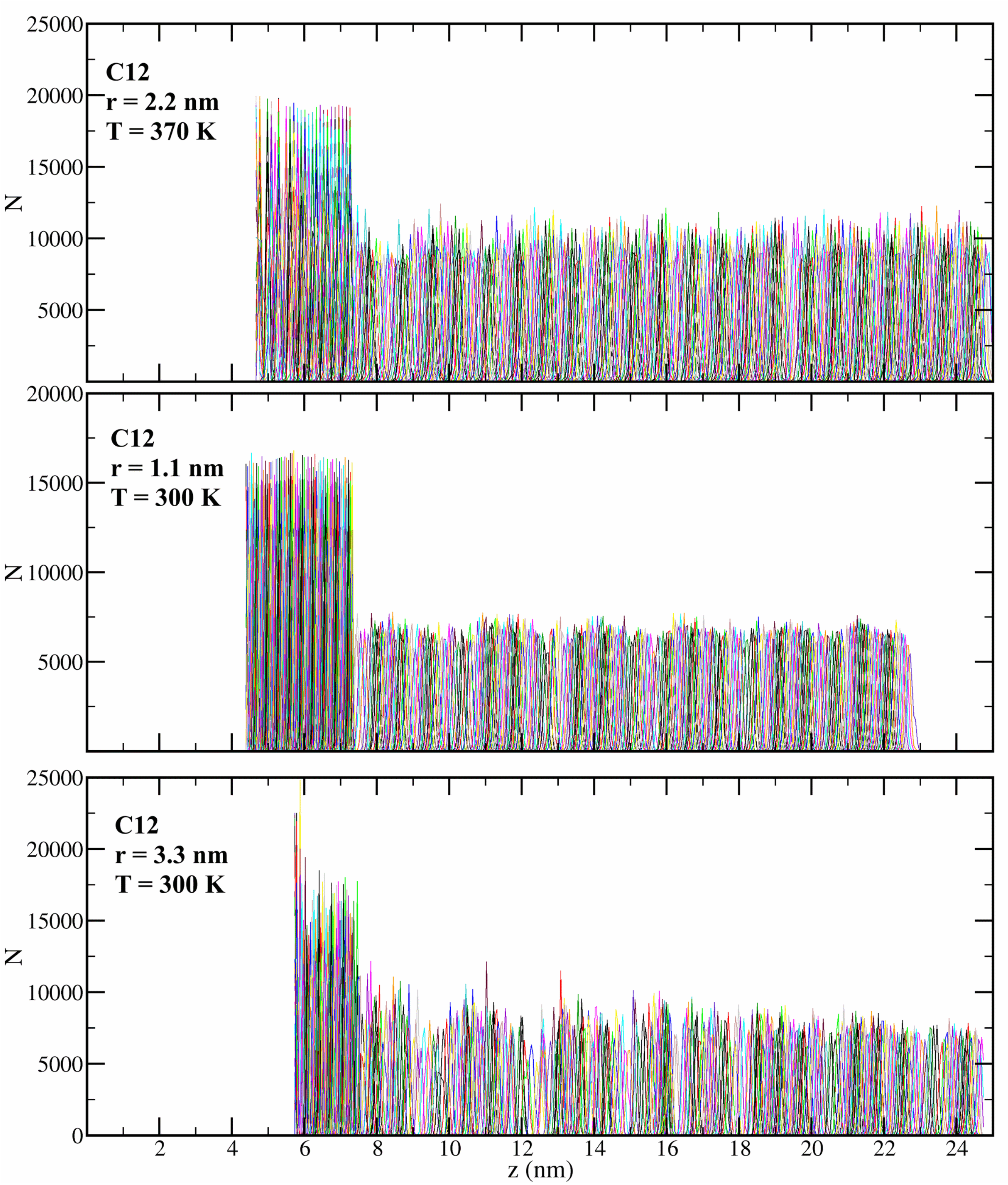

Figure S3 - Histograms showing the sampled values of the position z of the NP center of mass at each sampling window of the additional potential of mean force calculations performed for C12 liquid.

4. Zoom over the pmfs for the C12 liquid

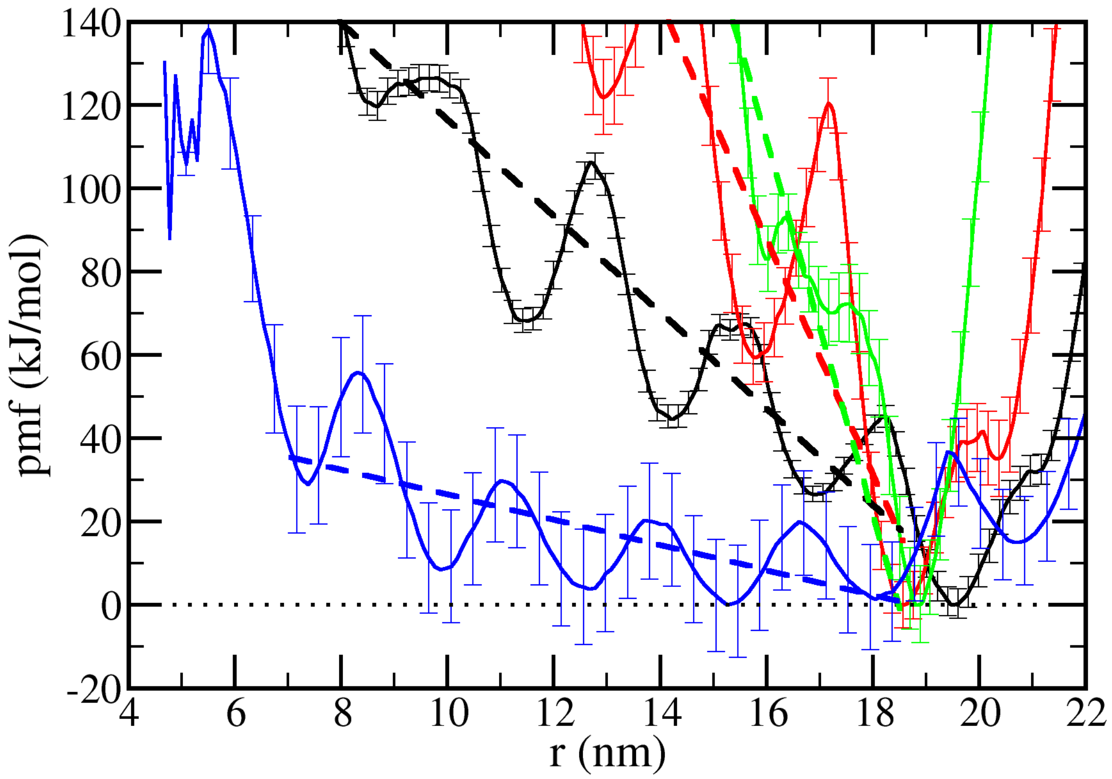

Figure S4 – Zoom over the pmfs computed for the C12 liquid (Figure 3 a of the manuscript) highlighting the regions of smaller free energy values, specially to enable a better view of the pmf at T=370 K temperature. Colors: Black: NP radius 1.1 nm and T = 300 K; red: NP radius 2.2 nm and T = 300 K; green: NP radius 3.3 nm and T = 300 K; and blue: NP radius 2.2 nm and T = 370 K. The dashed lines shows the linear regression performed between z = 7 and z = 18 nm.

5. Additional density profiles for C12 liquid

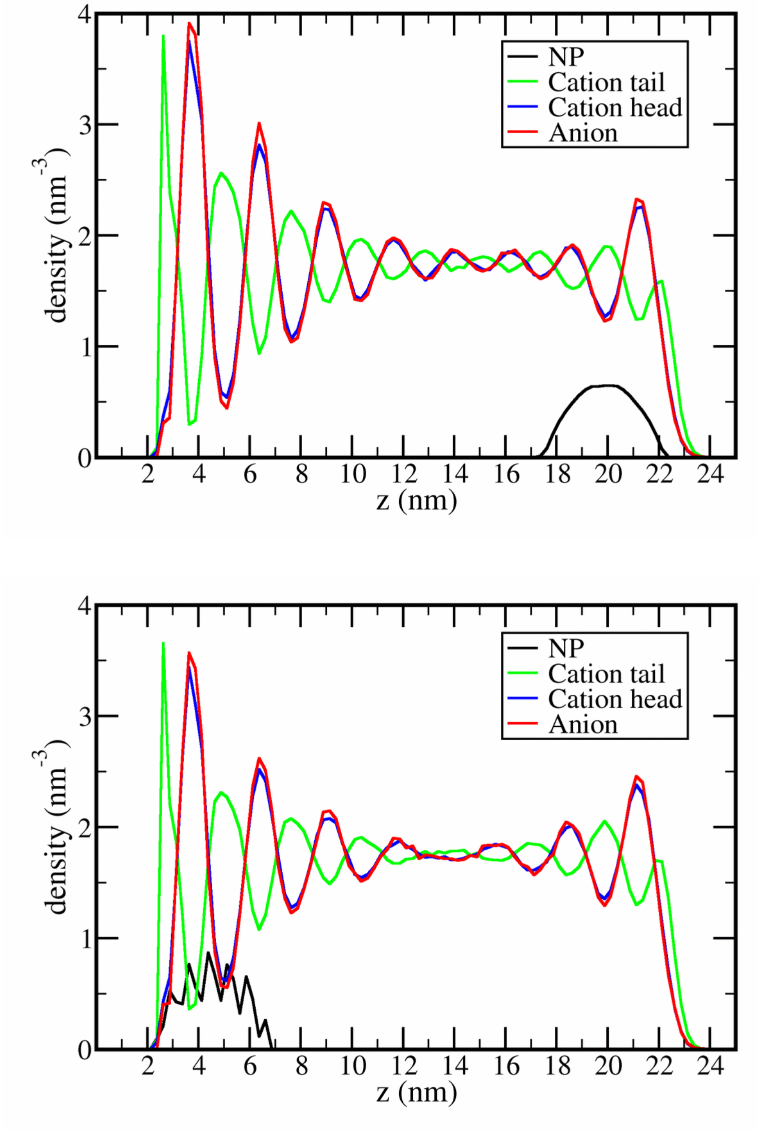

Figure S5 – Number density of NP and IL sites in the direction perpendicular to the solid surface in the simulations corresponding to the pmf minimum at liquid/vacuum interface (top) and in the simulation corresponding to the minimum at the contact with the surface (bottom) for the C12 liquid at 370 K.
